# Relation between proteome characterization and semen quality in Italian chicken breeds

**DOI:** 10.1371/journal.pone.0333802

**Published:** 2025-10-08

**Authors:** Simona Nonnis, Elisa Maffioli, Joshua Grana, Manuela Madeddu, Luisa Zaniboni, Stefano Paolo Marelli, Vera Perricone, Nicolaia Iaffaldano, Gabriella Tedeschi, Silvia Cerolini

**Affiliations:** 1 Department of Veterinary Medicine and Animal Sciences, University of Milan, Via dell’Università 6, Lodi, Italy; 2 CRC Innovation for Well-Being and Environment (I-WE), University of Milan, Milan, Italy; 3 Department of Agricultural, Environmental and Food Science, University of Molise, Campobasso, Italy; University of Tehran, IRAN, ISLAMIC REPUBLIC OF

## Abstract

The conservation of local chicken breeds is essential to safeguard genetic biodiversity and promote sustainable poultry production. Sperm cryopreservation is a key tool for the long-term maintenance of genetic diversity by enabling the storage of male gametes from endangered or valuable breeders for future use in conservation and breeding programs. However, significant variability in semen quality and fertility across breeds limits the effectiveness of cryopreservation protocols. This study aimed to explore the relationship between sperm protein composition and semen quality in five Italian chicken breeds, Bionda Piemontese (BP), Bianca di Saluzzo (BS), Mericanel della Brianza (MB), Pepoi (Pe), and Robusta Maculata (R), known for distinct reproductive traits. Semen samples were analyzed for volume, concentration, membrane integrity, and sperm motility, and for sperm proteomes by a label-free shotgun proteomics approach, to characterize potential molecular pathways associated with semen quality. Significant inter-breed differences were observed in semen parameters; Pe roosters showed the highest semen volume and concentration, but lower values in kinematic traits, including curvilinear velocity (VCL), straight-line velocity (VSL), and average path velocity (VAP). RM and MB exhibited the most favorable sperm membrane integrity, progressive sperm motility and sperm kinematic profile, with high VCL, VSL, VAP, linearity and wobble, despite lower semen volume. The remaining breeds showed intermediate values across most traits. Overall, results suggest breed-specific patterns and a potential trade-off between semen quantity and sperm motion efficiency. Proteomic analysis showed that proteins involved in energy metabolism, cytoskeletal dynamics, and membrane integrity were differentially abundant across breeds and correlated with specific semen traits. Gene Set Enrichment Analysis revealed enrichment of pathways such as the HSP90 chaperone cycle, TP53 transcriptional regulation, and insulin-like growth factor signaling in association with sperm motility and quality. Our findings demonstrate that sperm proteomes are associated with breed-specific fertility traits. This study provides new insights into the molecular basis of semen quality variability and biological conservation of avian genetic resources.

## Introduction

Conservation of animal genetic resources is globally recognized as essential for maintaining biodiversity and meeting the future needs of livestock production, which are strongly influenced by environmental change, sustainability, and animal welfare [1]. In the poultry sector, biodiversity conservation is particularly important due to the widespread reliance on highly productive commercial strains, which threatens the survival of local breeds [2]. According to FAO global data, 50% of known breeds of poultry are classified at risk of extinction in Europe [3] and conservation strategies and techniques have been developed and implemented over the years to decrease the risk of further losses. Studies on genetic diversity [4,5] and reproductive technologies [6–9] have been developed to support *in situ* and *ex situ in vitro* techniques [10] for conservation of local chicken populations.

Sperm cryopreservation is the major implemented reproductive technique for *ex situ in vitro* storage of avian genetic diversity; however, the fertilizing ability of cryopreserved semen is still very variable [9]. Over the last decades, research has mainly focused on cryoprotectants and *in vitro* protocols to improve frozen/thawed semen fertility; however, the mechanisms behind the loss of fertilizing ability in chicken sperm after cryopreservation remain unknown. A comprehensive approach including conventional, molecular and cellular studies is considered a key breakthrough to characterize peculiar semen composition and functions at the basis of male fertility and their changes during storage [7]. Variability in sensitivity to sperm cryopreservation was found among chicken breeds/lines [11–13], and among birds within breed [14], despite concomitant variability in *in vitro* semen quality was not always present. A few studies have suggested a relationship between the quality of fresh semen and the *in vivo* fertilizing ability of cryopreserved semen, whereas no such relationship has been observed when semen quality is assessed after freezing/thawing [14,15].

Deeper investigations characterizing sperm and seminal plasma at the molecular level in different chicken breeds/lines may provide valuable insights into the biological functions underlying variability in fertilizing ability and semen storability. Among various technical approaches, proteomics is considered particularly well-suited to investigate the molecular features of male fertility [16]. In chickens, recent studies on sperm and seminal plasma proteomes have revealed breed/line-specific compositions associated with semen quality and fertility [17].

In this context, recent studies have demonstrated that the seminal proteome varies significantly among individuals and breeds, directly influencing sperm fertilizing ability. Proteomic analysis allows the identification of biomarkers associated with fertility, such as enzymes involved in energy metabolism, respiratory chain or oxido-reduction activity, which play a crucial role in sperm survival and motility [18].

Furthermore, new studies have combined complementary proteomic strategies, such as Intact Cell MALDI-TOF Mass Spectrometry and GeLC-MS/MS, to maximize the identification of proteins related to male fertility [19]. These analyses revealed 57 differentially abundant proteins between fertile and subfertile roosters, highlighting key molecular pathways involved in flagellum integrity and movement, mitochondrial function, sperm maturation, storage in the female reproductive tract, and oocyte–sperm interaction. These findings emphasize the complexity of male fertility regulation and suggest that a combination of molecular markers could be a promising approach to better predict fertility outcomes in roosters.

Advanced mass spectrometry and quantitative analysis techniques, such as liquid chromatography coupled with mass spectrometry (LC-MS/MS), have enabled detailed characterization of the sperm proteome and the identification of key proteins for cryopreservation resistance [20]. This approach offers new perspectives for improving conservation strategies of local breeds and optimizing avian sperm cryopreservation techniques.

The aim of the present study was to characterize the sperm proteome in chicken Italian breeds with different quantitative and qualitative sperm production to study proteome variability and the association with sperm functions.

## Materials and methods

### Bird management

Adult chicken male breeders of five Italian breeds were housed indoors in individual cages at the Poultry Unit, Animal Production Centre, Veterinary Campus (University of Milan, Lodi). The Italian breeds were: *Bionda Piemontese* (BP, N = 19), *Bianca di Saluzzo* (BS, N = 18), *Mericanel della Brianza* (MB, N = 24), *Pepoi* (Pe, N = 15), *Robusta Maculata* (RM, N = 17). Birds were reared in controlled environmental conditions (T 20 °C; RU 60%; photoperiod 14L:10D), fed *ad libitum* a standard commercial breeder diet (2800 kcal ME/kg, 15% CP) and fresh drinking water. Birds were monitored twice daily by trained personnel to assess their health status. Bird handling was in accordance with the Guidelines for the Care and Use of Agricultural Animals in Research and Teaching [21]. The experimental protocol was approved by the Animal Welfare Committee (OPBA) of the University of Milan (authorization OPBA_103_2020).

### Semen collection

Semen was collected using the abdominal massage and milking method described by Burrows and Quinn [22]. Two trained operators conducted the procedure. The bird was placed on its breast and its legs restrained by one operator, while the other operator induced the phallus tumescence by massage of the male’s abdomen, and stroking the back and tail feathers; then, ejaculation was caused by gentle cloacal squeezing and semen collected directly into glass tubes. No anesthesia was used.

To ensure consistency, semen collection was always performed by the same operator at the same time of day, between 09:00 and 11:00 a.m. All males were of similar age, approximately one year old at the time of sampling. The average body weights (mean ± SD) for each breed were as follows: MB = 1079 ± 115 g, BP = 3283 ± 281 g, BS = 2960 ± 132 g, Pe = 1957 ± 210 g, and RM = 4167 ± 413 g.

Birds were trained for semen collection for a few weeks and semen donors who provided ejaculates without contaminants (urine, lymph, feces) were identified. Then, ejaculates were routinely collected twice weekly from semen donors (BP, N = 13; BS, N = 11; MB, N = 8; Pe, N = 15; RM, N = 12) for two months (2 collections/week per 2 months = 16 ejaculates/bird) in order to collect the required amount of samples and replicates for assessment of semen quality and biochemical analyses.

### Semen management

**Fig 1:**
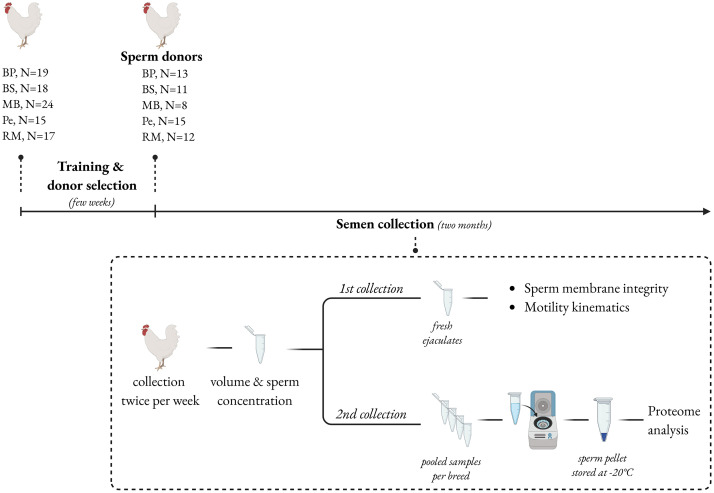
Illustration of the experimental design for semen collection, processing and analyses (created with BioRender.com).

Ejaculates were collected into 2.5 mL conical glass graduated tubes and volume (Vo, mL) was immediately recorded. Sperm concentration (Co, X 10^9^/mL) was assessed by colorimetric technique with a calibrated photometer (Accucell Photometer, IMV Technologies, L’Aigle, France) at a standard wavelength (535 nm) for fowl semen. On one day of weekly collection, semen quality was assessed on ejaculates by measuring sperm membrane integrity (SMI) and sperm motion parameters. On the other day of weekly collection, ejaculates within breed were pooled in semen samples for proteome analyses. Soon after collection, ejaculates were pooled in order to have at least 1 X 10^9^ sperm/mL, diluted (1:2) in 7.1 Lake extender (6 g D-glucose, 1.28 g potassium citrate, 15.2 g sodium glutamate, 0.8 g magnesium acetate; 30.5 g BES, 58 mL 1M sodium hydroxide, adjusted to 1 L with distilled water; pH 7.1 and osmolality 410 mOsm/kg) [23] at room temperature (i.e., 20°C) and centrifuged at 950x*g* for 20 min at 20°C; sperm pellet was further centrifuged at 1900x*g* for 20 min at 5°C. The final sperm pellet was stored at −20°C until proteome analyses. According to the amount of the ejaculates produced the following pooled semen samples were processed: BP, N = 9; BS, N = 9; MB, N = 4; Pe, N = 10; RM, N = 5. A summary of the experimental design is presented in Fig 1.

### Sperm motility kinematics

SMI and sperm motion variables were assessed in fresh ejaculates for each bird. SMI was assessed using the SYBR-14/propidium iodide fluorescent assay (LIVE/DEAD Sperm Viability Kit, Molecular Probes®, Invitrogen, Carlsbad, CA), as reported by Mosca et al. (2019) [24]. In brief, 5 μL semen were incubated with 50 μL SYBR14 solution for 10 min and then 5 μL PI solution added for further 5 min at room temperature. Assessment of 200 sperm was made in duplicate aliquots for every sample and evaluated microscopically at 1000 X total magnification using a Zeiss (Axioskop 40- AxioCamICc 1) microscope and FITC filter fluorescence. Sperm motility kinematics were assayed at room temperature using the computer-aided sperm analysis (CASA) system Sperm Class Analyzer (SCA) (version 4.0, Microptic S.L., Barcelona, Spain) coupled to a phase contrast microscope (Nikon Eclipse model 50i; negative contrast). Ten μL of fresh ejaculate diluted to 50 X 10^6^ sperm/mL in 0.9% NaCl were placed on a Makler chamber (Sefi Medical Instruments, Haifa, Israel) and assessed under microscope at room temperature. The following motion variables were recorded: total motile sperm (TMS, %), progressive motile sperm (PMS, %), curvilinear velocity (VCL, μm/s), straight-line velocity (VSL, μm/s), average path velocity (VAP, μm/s), amplitude of lateral head displacement (ALH, μm), beat cross frequency (BCF, Hz); the following velocity ratios were also calculated: linearity (LIN) = VSL/VCL X 100 (%), straightness (STR) = VSL/VAP X 100 (%), wobble (WOB) = VAP/VCL X 100 (%). A minimum of three fields and 500 sperm tracks were analyzed at 100 X magnification for each sample. The SCA setting provided for chicken semen was used: cell size from 5 to 190 µm^2^; frame rate (fps) = 25; motile (VCL) ≥ 13 μm/s; static (VCL) < 13 μm/s; rapid (VCL) > 100 μm/s, progressive (STR) ≥ 70, connectivity (pixels) = 18.

### Proteomic analysis by a shotgun label free approach

Final sperm pellets were analyzed by a shotgun label free proteomic approach for the identification and quantification of the detected proteins. Samples were suspended in urea 8 M/Hepes 20 mM pH 8.0 containing protease inhibitors cocktail (Roche), sonicated using an ultrasonic probe in bursts of 60 s on ice and centrifuged at 16,560 × g for 15 min at 16 °C to pellet the tissue debris as previously reported [25]. The protein content was determined by the Bradford assay with bovine serum albumin as standard. Prior to proteolysis, proteins were reduced with 13 mM dithioerythritol (DTE; 30 min at 56 °C) and alkylated with 26 mM iodoacetamide (IAA; 30 min at room temperature, in the dark). Protein digestion was performed using sequence-grade trypsin (Promega) for 16 h at 37 °C using a protein: enzyme ratio of 20:1. The collected peptides were desalted using Zip-Tip C18 before mass spectrometric (MS) analysis [26, 27]. NanoHPLC coupled to MS/MS analysis was performed on Dionex UltiMate 3000 directly connected to an Orbitrap Fusion Tribrid mass spectrometer (Thermo Fisher Scientific, Waltham, MA, USA) by a nanoelectrospray ion source. Peptide mixtures were enriched on 75 μm ID × 150 mm EASY Spray PepMap RSLC C18 column (Thermo Fisher Scientific) and separated using the LC gradient: 4% ACN in 0.1% formic acid for 3 min, 4–28% ACN in 0.1% formic acid for 100 min, 28–40% ACN in 0.1% formic acid for 10 min, 40–95% ACN in 0.1% formic acid for 1 min and 95–4% ACN in 0.1% formic acid for 3 min at a flow rate of 0.3 μL/min. Orbitrap MS spectra of eluting peptides were collected over an m/z range of 375–1500 at resolution of 120,000, operating in a data-dependent mode with a cycle time of 3 s between master scans. HCD MS/MS spectra were acquired in Orbitrap at resolution of 15,000 using a normalized collision energy of 35%, and an isolation window of 1.6 m/z. Dynamic exclusion was set to 60 s. Rejection of +1 and unassigned charge states were enabled [28].

The mass spectrometry proteomics data have been deposited to the ProteomeXchange Consortium via the PRIDE [29] partner repository, with the dataset identifier PXD056416.

A database search was conducted against the Uniprot *Gallus gallus* database (released in 09/2022; 47,530 entries) with MaxQuant (version 1.6.1.0) software. The initial maximum allowed mass deviation was set to 10 ppm for monoisotopic precursor ions and 0.5 Da for MS/MS peaks. Enzyme specificity was set to trypsin, defined as C-terminal to Arg and Lys excluding Pro, and a maximum of two missed cleavages were allowed. Carbamidomethylcysteine was set as a fixed modification, while Met oxidation, Asn/Gln deamidation and N-terminal acetylation were set as variable modifications. Quantification in MaxQuant was performed using the built-in label free quantification algorithms (LFQ) based on extracted ion intensity of precursor ions. The false discovery rate (1%) of peptide-spectrum matches (PSMs) and protein identifications was estimated by searching MS/MS spectra against the corresponding reversed-sequence (decoy) database [30].

Parallel Reaction Monitoring (PRM) analysis was performed to validate few proteins based on their functional relevance in key pathways identified through enrichment analysis, namely dynactin subunit 5 (*DCTN5*), NFS attachment protein alpha (*NAPA*), sulfurtransferase (*MPST*), and glycerol-3-phosphate phosphatase (*PGP*).

PRM acquisition analytical conditions consisted of MS2 scans triggered by an inclusion list from either DCTN5, NAPA, MPST, and PGP peptides, without time scheduling, imported from Skyline software (https://skyline.ms/project/home/software/Skyline/begin.view).

MS2 scans were collected at a resolution of 35,000 with an AGC target value of 2 × 10^5^, a maximum injection time of 100 ms, and an isolation window of 1.5 m/z.

PRM raw data files were searched using Skyline software (version 25.1.0.142) against a protein sequence database of the four target proteins. Search parameters included: digestion by trypsin with a maximum of 2 missed cleavage sites, a minimum peptide length of 6, and a mass deviation of 20 ppm for monoisotopic precursor ions and 100 ppm for MS/MS peaks. Carbamidomethylcysteine was set as a fixed modification, while Asn/Gln deamidation were set as variable modifications.

### Statistical and bioinformatic analysis

Analysis of variance was performed on semen variables using the MIXED procedure of SAS version 9 (1999), followed by Tukey test for multiple comparison analysis of LSMeans; statistical significance was set at p-value < 0.05. The statistical model included the fixed effect ‘breed’ and the random effect ‘bird’.

The proteomic data analysis was performed using the Perseus software (version 1.5.5.3) [31]. Only the proteins present and quantified in at least 75% of the repeats were considered positively identified in a sample. Based on the number of identified proteins and Pearson’s coefficient values between the different datasets, four raw files from each group were chosen for further analyses. Principal Component Analysis (PCA) was carried out on quantitative data related to proteins in each group (MB, BP, BS, Pe and RM).

Bioinformatics analysis of proteins present exclusively in one condition was performed by ClueGo-Reactome software (Cytoskape release 3.10.1, ref. species *Gallus gallus*) [32] to cluster enriched annotation groups of Pathways within the set of the exclusively identified proteins.

Gene Set Enrichment Analysis (GSEA, release 4.3.2.) was carried out in order to better correlate semen properties with protein composition [33]. The five breeds were evaluated giving an arbitrary score based on the semen quality parameters: + 2 to the breeds with the highest performance, −2 for the lowest performance and 0 to the groups with intermediate properties. Breeds with the same score were clustered to generate, for each semen quality parameter, a group with score 2 (highest performance) and a group with score −2 (lowest performance). These groups were subsequently compared using GSEA, focusing on molecular pathways (Reactome) and biological processes (GOBP). Results were visualized by the Enrichment Map in Cytoskape (release 3.10.1) (p-value < 0.005).

Interaction networks were visualized using the “Search Tool for Recurring Instances of Neighbouring Genes” (STRING 12.0 release July 26, 2023) [34], setting the minimum required interaction score at 0.4 and hiding disconnected nodes.

## Results

### Semen quality variables

Semen production and quality were significantly different between breeds and mean values of sperm quality parameters are reported in Table 1. The highest quantitative semen production, according to volume and concentration mean values, was recorded in Pe breed and, in contrast, the lowest in RM breed; an intermediate similar semen production was recorded in BP, BS, and MB. The high sperm concentration of Pe semen was associated with a high proportion of sperm with undamaged membrane (SMI, 94%); similar high SMI proportions (96%) were also found in RM and MB semen, whereas significantly lower SMI proportions were found in BP and BS semen, 87% and 84% respectively. The proportion of TMS ranged from 83% to 92% according to the breed, and no significant differences were found between them. In contrast, the highest proportion of PMS was recorded in RM semen, progressively lower proportions were recorded in MB, Pe and BP semen, and the lowest proportion, in BS semen. VCL mean values ranged from 62 μm/s in Pe semen to 78 μm/s in MB semen; however, a significant difference was found only between RM and Pe mean values. The best VSL, LIN, STR and WOB mean values were recorded in RM ejaculates, being significantly higher compared to the mean values recorded in BP, BS and Pe ejaculates. VSL, VAP, LIN, STR, and WOB mean values recorded in MB semen were similar to those recorded in RM semen, and also numerically higher compared to BP, BS and Pe semen. The lowest STR value, 61%, was recorded in BS ejaculates, being significantly lower compared with RM and Pe mean values. Similarly, high ALH mean values were found in BS and BP semen, and significantly lower mean values were found in Pe and RM semen; ALH value in MB semen was similar to those of all other breeds. No significant differences were present among breeds for BCF (Hz).

**Table 1:** Sperm quality variables in Italian chicken breeders including Bionda Piemontese (BP, N = 13), Bianca di Saluzzo (BS, N = 11), Mericanel della Brianza (MB, N = 8), Pepoi (Pe, N = 15), and Robusta Maculata (RM, N = 12) evaluated weekley over a two months period. Values are Means ± SE.

Semen	Breed				
parameters^1^	BP	BS	MB	Pe	RM
Vo (mL)	0.32 ± 0.03 ab	0.29 ± 0.04 ab	0.11 ± 0.08 bc	0.39 ± 0.03 a	0.14 ± 0.04 c
Co (X 10^9^/mL)	2.87 ± 0.21b	3.00 ± 0.23 b	2.71 ± 0.49 bc	4.45 ± 0.17 a	1.80 ± 0.21 c
SMI (%)	86.84 ± 1.69 bc	84.35 ± 1.81 c	96.19 ± 3.74 ab	94.21 ± 1.36 a	96.11 ± 1.75 a
TMS (%)	84.52 ± 2.77	83.21 ± 3.09	92.46 ± 6.38	87.08 ± 2.25	88.20 ± 2.85
PMS (%)	22.27 ± 1.98 b	20.13 ± 2.24 b	30.06 ± 4.57 ab	24.11 ± 1.63 b	32.82 ± 2.05 a
VCL (μm/s)	70.60 ± 3.87 ab	69.10 ± 4.15 ab	77.63 ± 9.07 ab	62.41 ± 2.95 b	76.63 ± 3.96 a
VSL (μm/s)	27.14 ± 1.74 b	25.04 ± 1.93 b	33.28 ± 3.99 ab	25.28 ± 1.41 b	34.98 ± 1.78 a
VAP (μm/s)	43.56 ± 2.58 ab	41.83 ± 2.83 ab	51.58 ± 5.97 ab	39.80 ± 2.04 b	51.50 ± 2.64 a
LIN (%)	40.28 ± 1.09 b	37.54 ± 1.14 b	43.25 ± 2.59 ab	41.08 ± 0.79 b	47.23 ± 1.11 a
STR (%)	63.24 ± 0.87 bc	60.72 ± 0.89 c	64.52 ± 2.17 abc	64.13 ± 0.60 b	69.14 ± 0.89 a
WOB (%)	63.19 ± 0.88 b	61.44 ± 0.95 b	66.67 ± 2.03 ab	63.66 ± 0.68 b	67.59 ± 0.89 a
ALH (μm)	3.81 ± 0.10 a	3.78 ± 0.11 a	3.67 ± 0.25 ab	3.31 ± 0.07 b	3.27 ± 0.11 b
BCF (Hz)	7.02 ± 0.16	6.84 ± 0.18	7.69 ± 0.37	7.40 ± 0.13 c	6.95 ± 0.16

a-c Means within a row with different letters are significantly different with p-value < 0.05.

1 Vo, sperm volume; Co, sperm concentration; SMI, sperm membrane integrity; TMS, total motile sperm; PMS, progressive motile sperm; VCL, curvilinear velocity; VSL, straight-line velocity; VAP, average path velocity; LIN (VSL/VCL X 100), linearity; STR (VSL/VAP X 100), straightness; WOB (VAP/VCL X 100), wobble; ALH, amplitude of lateral head displacement; BCF, beat cross frequency.

### Mass spectrometry‑based proteomic analysis

**Fig 2:**
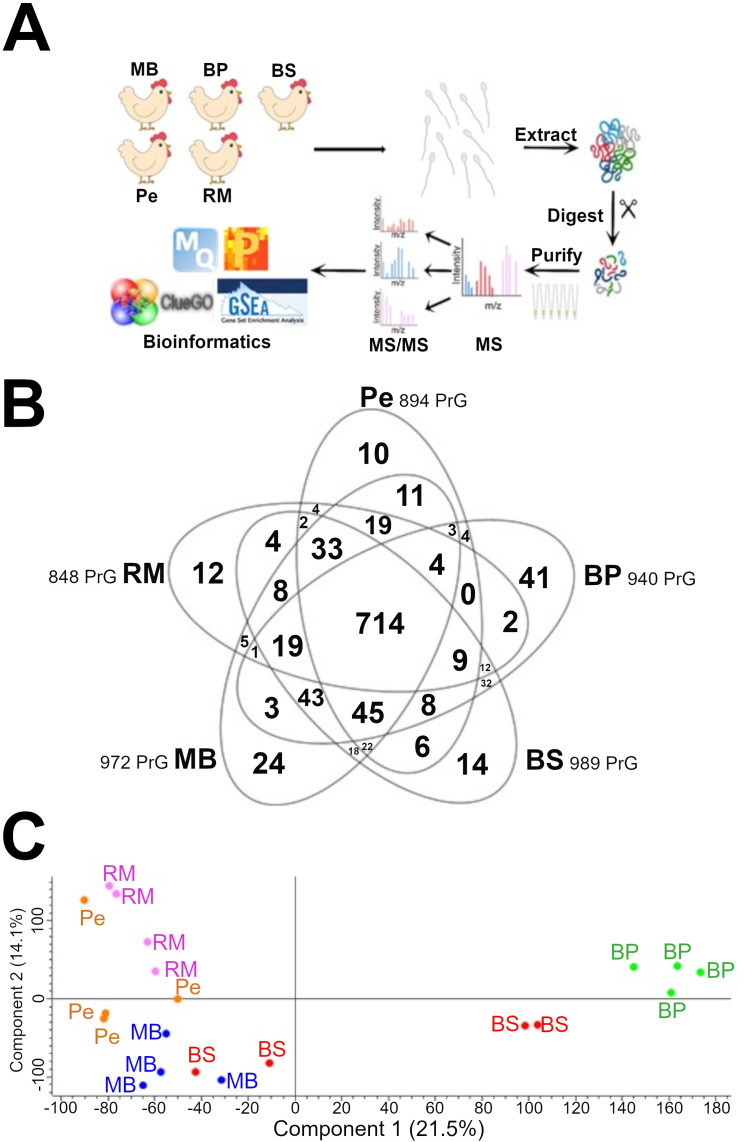
Shotgun label-free quantitative proteomic analysis. **A)** Overview of the workflow applied for the analysis of sperm proteome from Bionda Piemontese (BP), Bianca di Saluzzo (BS), Mericanel della Brianza (MB), Pepoi (Pe) and Robusta Maculata (RM) chicken breeds. **B)** Venn diagram of the proteins identified in the five data sets. The proteomic analysis allowed to identify proteins common to all samples, as well as proteins exclusively detected in each data set (indicated in bold). The term PrG = Protein Group. **C)** Principal Component Analysis (PCA).

Shotgun label-free proteomics allowed to identify 972, 940, 989, 894 and 848 proteins in MB, BP, BS, Pe and RM, respectively (Fig 2A, S1-S5 Tables).

The consistency of the protein extraction, the efficacy of the whole proteomics workflow, as well as the low sample variability within breeds, were confirmed by the evaluation of: (I) the number of identified proteins, (II) the percentage of MS/MS analyses performed, (III) the Pearson’s coefficient values between different datasets (S6 Table).

Datasets with the highest Pearson’s coefficient values (N = 4 per breed) were used for differential analysis. Venn diagram of all datasets shows both common and breed-specific proteins (Fig 2B). Principal Component Analysis (PCA) (Fig 2C) reveals clear clustering of datasets by breeds, except for BS and Pe which formed pairs.

### Bioinformatic analysis of the five chicken breeds proteomes

Breed-specific proteins (Fig 2B and S7-S11 Tables) were analyzed by ClueGo-Reactome to highlight the major differences between breeds in terms of molecular pathways (Table 2).

**Table 2:** Functional analysis by ClueGo-Reactome of the proteins exclusively present in the sperm of five Italian chicken breeds.

Proteins exclusively present in BP					
Pathway	p-value	FDR	genes	proteins	references
Surfactant metabolism	2.67E-05	8.05E-03	PSAP	Prosaposin	[45,54]
Defective CSF2RA causes SMDP4	3.27E-04	3.27E-02	PSAP	Prosaposin	[45,54]
Defective CSF2RB causes SMDP5	3.27E-04	3.27E-02	PSAP	Prosaposin	[45,54]
Diseases associated with surfactant metabolism	1.29E-03	8.90E-02	PSAP	Prosaposin	[45,54]
Signal regulatory protein family interactions	1.62E-03	8.90E-02	PSAP	Prosaposin	[45,54]
TICAM1, RIP1-mediated IKK complex recruitment	1.80E-03	8.90E-02	TFAM	Transcription factor A, mitochondrial	[47,48,55]
			UBE2V1	Ubiquitin-conjugating enzyme E2 variant 1 (UEV-1) (CROC-1B)	[46]
Toll Like Receptor 4 (TLR4) Cascade	2.07E-03	8.90E-02	TFAM	Transcription factor A, mitochondrial	[47,48,55]
			UBE2V1	Ubiquitin-conjugating enzyme E2 variant 1 (UEV-1) (CROC-1B)	[46]
			PSAP	Prosaposin	[45,54]
**Proteins exclusively present in BS**					
Pathway	pValue	FDR	genes	proteins	references
RAB GEFs exchange GTP for GDP on RABs	5.83E-03	1.40E-01	GDI1	Rab GDP dissociation inhibitor	
Rab regulation of trafficking	1.06E-02	1.40E-01	GDI1	Rab GDP dissociation inhibitor	
Membrane Trafficking	4.49E-02	1.52E-01	PPP6C	Serine/threonine-protein phosphatase	[56]
			GDI1	Rab GDP dissociation inhibitor	
Terminal pathway of complement	1.02E-02	1.40E-01	C8B	Complement component C8 beta chain	[57]
Receptor Mediated Mitophagy	1.40E-02	1.40E-01	FUNDC1	FUN14 domain containing 1	[58]
Interaction With Cumulus Cells And The Zona Pellucida	1.40E-02	1.40E-01	ADAM20	ADAM metallopeptidase domain 20	
Telomere Extension By Telomerase	2.91E-02	1.52E-01	PPP6C	Serine/threonine-protein phosphatase	[56]
Fertilization	3.29E-02	1.52E-01	ADAM20	ADAM metallopeptidase domain 20	[59]
Mitophagy	3.66E-02	1.52E-01	FUNDC1	FUN14 domain containing 1	[58]
Synthesis of active ubiquitin: roles of E1 and E2 enzymes	3.78E-02	1.52E-01	UCHL3	Ubiquitin carboxyl-terminal hydrolase (EC 3.4.19.12)	[44]
**Proteins exclusively present in MB**					
Pathway	pValue	FDR	genes	proteins	references
Digestion of dietary carbohydrate	3.54E-04	5.30E-02	AMY2A	Alpha-amylase	
Glutamate and glutamine metabolism	6.54E-04	5.30E-02	GLUD2	glutamate dehydrogenase [NAD(P)(+)], 1.4.1.3	[60]
Metabolism of amino acids and derivatives	2.15E-03	6.70E-02	SLC25A21	Solute carrier family 25 member 21	[61]
			SLC25A15	Solute carrier family 25 member 15	[62,63]
			GLUD2	glutamate dehydrogenase [NAD(P)(+)], 1.4.1.3	[60]
			RPS8	40S ribosomal protein S8	[39]
Digestion and absorption	2.24E-03	6.70E-02	AMY2A	Alpha-amylase	
Transcriptional activation of mitochondrial biogeneis	8.86E-03	2.04E-01	GLUD2	glutamate dehydrogenase [NAD(P)(+)], 1.4.1.3	[60]
Peptide hormone metabolism	2.01E-02	2.42E-01	ACE	Angiotensin-converting enzyme, ACE, 3.4.15.1, Dipeptidyl carboxypeptidase I, Kininase II	[64]
Mitochondrial biogeneis	2.37E-02	2.42E-01	GLUD2	glutamate dehydrogenase [NAD(P)(+)], 1.4.1.3	[60]
Organelle biogenesis and maintenance	3.67E-02	2.42E-01	ARL6	ADP-ribosylation factor-like protein 6	[65]
			GLUD2	glutamate dehydrogenase [NAD(P)(+)], 1.4.1.3	[60]
Defective SLC26A3 causes congenital secretory chloride diarrhea 1 (DIAR1)	2.48E-03	6.70E-02	SLC26A3	Chloride anion exchanger	[66]
Calcineurin activates NFAT	2.21E-02	2.42E-01	PPP3CA	Serine/threonine-protein phosphatase, 3.1.3.16	[40]
Multifunctional anion exchangers	2.21E-02	2.42E-01	SLC26A3	Chloride anion exchanger	[66]
Urea cycle	2.45E-02	2.42E-01	SLC25A15	Solute carrier family 25 member 15	[62,63]
Neurotransmitter clearance	2.45E-02	2.42E-01	ACE	Angiotensin-converting enzyme, ACE, 3.4.15.1, Dipeptidyl carboxypeptidase I, Kininase II	[64]
CLEC7A (Dectin-1) induces NFAT activation	2.70E-02	2.42E-01	PPP3CA	Serine/threonine-protein phosphatase, 3.1.3.16	[40]
Lysine catabolism	2.94E-02	2.42E-01	SLC25A21	Solute carrier family 25 member 21	[61]
Synthesis of IP2, IP, and Ins in the cytosol	3.42E-02	2.42E-01	INPP5J	Inositol polyphosphate-5-phosphatase J	
Metabolism of Angiotensinogen to Angiotensins	4.37E-02	2.42E-01	ACE	Angiotensin-converting enzyme, ACE, 3.4.15.1, Dipeptidyl carboxypeptidase I, Kininase II	[64]
Synthesis, secretion, and deacylation of Ghrelin	4.85E-02	2.42E-01	ACE	Angiotensin-converting enzyme, ACE, 3.4.15.1, Dipeptidyl carboxypeptidase I, Kininase II	[64]
**Proteins exclusively present in Pe**					
Pathway	pValue	FDR	genes	proteins	references
Beta-oxidation of pristanoyl-CoA	7.68E-03	1.04E-01	CRAT	Carnitine O-acetyltransferase	[42]
CREB1 phosphorylation through the activation of Adenylate Cyclase	1.02E-02	1.04E-01	PRKX	Protein kinase, X-linked	[40,67]
PKA-mediated phosphorylation of CREB	1.70E-02	1.04E-01	PRKX	Protein kinase, X-linked	[40,67]
Peroxisomal lipid metabolism	2.46E-02	1.04E-01	CRAT	Carnitine O-acetyltransferase	[42]
Calmodulin induced events	2.96E-02	1.04E-01	PRKX	Protein kinase, X-linked	[43,68]
CaM pathway	2.96E-02	1.04E-01	PRKX	Protein kinase, X-linked	[43,68]
Ca-dependent events	3.12E-02	1.04E-01	PRKX	Protein kinase, X-linked	[43,68]
DAG and IP3 signaling	3.46E-02	1.04E-01	PRKX	Protein kinase, X-linked	[43,68]
ADORA2B mediated anti-inflammatory cytokines production	3.70E-02	1.04E-01	PRKX	Protein kinase, X-linked	[43,68]
PLC beta mediated events	4.12E-02	1.04E-01	PRKX	Protein kinase, X-linked	[43,68]
G-protein mediated events	4.53E-02	1.04E-01	PRKX	Protein kinase, X-linked	[43,68]
**Proteins exclusively present in RM**					
Pathway	pValue	FDR	genes	proteins	references
Regulation of gene expression in beta cells	3.38E-04	1.45E-02	PKLR	Pyruvate kinase	[35]
Regulation of beta-cell development	1.22E-03	2.56E-02	PKLR	Pyruvate kinase	[35]
Glycolysis	3.34E-03	4.68E-02	PKLR	Pyruvate kinase	[35]
Glucose metabolism	5.38E-03	5.38E-02	PKLR	Pyruvate kinase	[35]
Metabolism of carbohydrates	4.91E-02	1.21E-01	PKLR	Pyruvate kinase	[35]
ChREBP activates metabolic gene expression	7.01E-03	5.61E-02	PKLR	Pyruvate kinase	[35]
Telomere C-strand synthesis initiation	1.47E-02	1.03E-01	TMEM107	Transmembrane protein 107	[36]
Polymerase switching on the C-strand of the telomere	2.40E-02	1.21E-01	TMEM107	Transmembrane protein 107	[36]
Telomere C-strand (Lagging Strand) Synthesis	3.76E-02	1.21E-01	TMEM107	Transmembrane protein 107	[36]
COPI-independent Golgi-to-ER retrograde traffic	4.81E-02	1.21E-01	DCTN5	Dynactin subunit 5	[37,38]

The proteins exclusively detected in each data set were analyzed by ClueGo-Reactome (Cytoskape release 3.10.1), p-value < 0.005, GO terms fusion allowed. For each protein identified, the table reports the main references in relation to male fertility and semen quality. Number of semen samples analyzed: BP, N = 4; BS, N = 4; MB, N = 4; Pe, N = 4; RM, N = 4.

In the RM breed, proteins involved in energy metabolism and sperm motility were identified, including PKLR, TMEM107 and DCTN5 (S11 Table). These proteins are functionally linked to glucose metabolism, sperm flagellum morphology, and spermatid growth [35–38].

Similarly, in the MB breed, the detection of proteins such as RPS8 and PPP3CA, together with others involved in mitochondrial membrane transport, suggests an active role in processes that are essential for sperm development and motility [39,40]. Additionally NAPA, a key protein in vesicle trafficking and Golgi/ER transport, was exclusively identified in MB. This protein also plays a role in head and central nervous system development pathways, further suggesting its involvement in the regulation of sperm structure and functionality in this breed (S7 Table) [41].

The Pe breed exhibits proteins such as Carnitine O-acetyltransferase (CRAT) and Protein Kinase X-linked (PRKX), involved in maintaining chromatin quality, cell adhesion, and endothelial cell proliferation, all of which are linked to membrane fluidity and overall integrity (S10 Table) [42,43]. For the BS breed, UCHL3, a protein involved in sperm motility and fertilization process was identified (S9 Table) [44]. In the BP breed, the proteins PSAP, TFAM and UBE2V1 were identified (S8 Table). These proteins are known to be involved in glycosphingolipid metabolism, mitochondrial function and protein degradation during spermatogenesis, respectively [45–49]. MPST, a sulfurtransferase implicated in central nervous system development and in maintaining redox homeostasis, was also exclusively detected in BP [50,51].

In accordance, the identified proteins have already been reported in the literature in various species, where they have been associated with semen quality parameters as reported in Table 3.

**Table 3:** Lists of proteins identified in the present study that have been previously reported from human, chicken, bovine or other species, namely mouse, Drosophila, and *in vitro* experiments.

Protein	Human	Chicken	Bovine	Other species
PKLR				[35,36]
TMEM107				[36]
DCTN5				[38]
RPS8	[39]			
PPP3CA				[40]
CRAT				[42]
PRKX	[43]			
UCHL3	[44]			
PSAP				[45]
TFAM	[47]			[48]
UBE2V1	[46]			
DYNLL	[69,52]	[18]	[53]	
DYNLL2	[69,52]	[18]	[53]	
TUBA1A	[69]	[18]	[53]	
TUBA3E	[69]	[18]	[53]	
TUBA4A	[69]	[18]	[53]	
TUBA2A	[69]	[18]	[53]	
TUBA4B	[69]	[18]	[53]	
CAPZA3	[52,70]		[55,53]	
CENPE				[71,72]
KIF9				[72]
KIF2B	[73]			
PAFAH1B1	[73]			
MT-CO1	[74]			
YWHAB		[20,75]		
YWHAE		[20,75,76]		
YWHAG		[20,75]		
YWHAH		[20,75]		
YWHAQ		[20,75]		
YWHAZ		[20,75]		
IGF-I	[77]		[78,79]	
IGF-II	[77]		[78,79]	
IGFBP-2	[77]		[78,79]	
CKB	[77]	[19]		
TF		[19]		
TCP1		[19]		
APOA1		[18,19,80]		
ENO1		[80]	[53,81]	
FGA		[80]		
MDH1		[18,80]	[82]	
PGAM1		[18,80]	[55]	
PGK2		[80]	[55]	
TPI1		[80]		
ACR		[18,83]		
GAPDH		[80]		

References to the relevant studies that identified/investigated each protein and the respective species, are indicated.

A scoring system based on the semen quality variables (+2 for the highest, −2 for the lowest and 0 for intermediate values) was applied to each breed. The final ranking of the breeds for each semen variable is reported in Table 4. Based on these ranking, proteomic datasets were clustered and analyzed using GSEA to identify enriched molecular pathways (Reactome) and biological processes (GOBP), reported in Table 5.

**Table 4:** Clustering of sperm proteomes based on semen quality parameters in Italian chicken breeds.

Semen variables	BP	BS	MB	Pe	RM	Comparisons
Vo (mL)	0	0	0	2	−2	(Pe) vs (RM)
Co (X 10^9^/mL)	0	0	0	2	−2	(Pe) vs (RM)
SMI (%)	0	−2	0	2	2	(Pe + RM) vs (BS)
PMS (%)	−2	−2	0	−2	2	(RM) vs (BP + BS + Pe)
VCL (μm/s)	0	0	0	−2	2	(RM) vs (Pe)
VSL (μm/s)	−2	−2	0	−2	2	(RM) vs (Pe + BS + BP)
VAP (μm/s)	0	0	0	−2	2	(RM) vs (Pe)
LIN (VSL/VCL)	−2	−2	0	−2	2	(RM) vs (Pe + BS + BP)
STR (VSL/VAP)	0	−2	0	0	2	(RM) vs (BS)
ALH (μm)	2	2	0	−2	−2	(BP + BS) vs (Pe + RM)
WOB (VAP/VCL)	−2	−2	0	−2	2	(RM) vs (BP + BS + Pe)

The score +2 (red), −2 (blue) and 0 was given to the breed with the highest, lowest and intermediate value, respectively, for each semen variable. The column “comparisons” indicates for each quality variable which breed clusters were compared by GSEA. Vo, sperm volume; Co, sperm concentration; SMI, sperm membrane integrity; PMS, progressive motile sperm; VCL, curvilinear velocity; VSL, straight-line velocity; VAP, average path velocity; LIN (VSL/VCL X 100), linearity; STR (VSL/VAP X x 100), straightness; WOB (VAP/VCL X 100), wobble; ALH, amplitude of lateral head displacement.Number of semen samples analyzed: BP, N = 4; BS, N = 4; MB, N = 4; Pe, N = 4; RM, N = 4.

**Table 5:** Gene Set Enrichment Analysis (GSEA) of Sperm Quality-Associated Proteins Across Breeds.

Sperm quality parameters (Comparison)	Enrichment map p-value	Genes	GSEA Biological Processes (GOBP)	GSEA Pathways (REACTOME)
WOB (RM) vs (BP + BS + Pe)	p = 0.05	ACTR1A, CAPZA3, CAPZB, DCTN5, DNAJA2, DNAJA4, DYNLL1, DYNLL2, FKBP4, HSP90AA1, HSPA8, PTGES3, STIP1, TUBA1A, TUBA3E, TUBA4A, TUBB2A, TUBB4B		HPS90 chaperone cycle for steroid hormone receptors SHR in the presence of ligand
ALH (BP + BS) vs (Pe + RM)	p = 0.005	TUBB3, ACTR1A, DNAJA4, TUBB2A, CAPZB, TUBA1A, DNAJA2, CAPZA3, TUBB4B, HSPA8, TUBA4A, HSP90AA1, PTGES3, TUBA3E, STIP1, DYNLL1, DYNLL2, FKBP4, DCTN5		HSP90 chaperone cycle for steroid hormone receptors SHR in the presence of ligand
Co (Pe) vs (RM)	p = 0.005	NELL2, KLHL10, PARK7, HEXB, CCT3, ACR, WBP2NL, PLCZ1, CCT2, TCP1, CCT8, CCT5, VDAC2, CCT7, CFAP69, ZPBP2, PRSS55, ASTL	Fertilization	
PMS (RM) vs (BP + BS + Pe)	p = 0.05	DCTN5, FKBP4, DYNLL2, STIP1, DYNLL1, PTGES3, HSP90AA1, TUBB4B, TUBA1A, HSPA8, TUBA4A, TUBA3E, CAPZA3, CAPZB, DNAJA2, DNAJA4, TUBB2A, ACTR1A		HSP90 chaperone cycle for steroid hormone receptors SHR in the presence of ligand
VAP (RM) vs (Pe)	p = 0.05	MSLN, AHSG, FN1, FGG, CKAP4, FGA, C3, SERPINC1, APOA1, PLG, F2, TF, ALB, HSP90B1, P4HB		Regulation of insulin like growth factor IGF transport and uptake by insulin like growth factor binding proteins IGFBPS
SMI (Pe + RM) vs (BS)	p = 0.01	CDC14B, CHP1, HDHD2, HSP90B1, IGBP1, LGALS3, MTMR9, PP2D1, PPEF1, PPIA, PPM1B, PPP1CA, PPP1CB, PPP1CC, PPP1R2, PPP1R7, PPP2CA, PPP2R5A, PPP6C, PTP4A2, PTPA, STYXL1, YWHAB, YWHAE	Dephosphorilation	
		C1QBP, CALR, CSNK1A1, DDX3X, FKBP4, HSPA8, PARK7, PDK3, PHB1, PHB2, PPP2CA, PTGES3, YWHAH	Intracellular receptor signaling pathway	
		ABAT, ACTB, AK8, ATP5PB, CDC42, CFAP43, CKB, DNAH5, DYNLL1, EFHC1, GAS8, HSPA5, HTRA2, HYDIN, MGARP, MKS1, MT-CO1, MT-ND4, NCSTN, NDUFS3, NDUFS4, NME5, PAFAH1B1, RAN, RRAS, SPEF2, TCTN1, TUBA1A, TUBB2A, UGP2, UQCRQ, WDR37, YWHAE, YWHAH, YWHA	Head development	
		ABAT, ACTB, AK8, ATP5PB, C3, CDC42, CFAP43, CKB, COX7B, DNAH5, DYNLL1, EFHC1, F2, GAS8, GSN, HSP90AA1, HSPA5, HTRA2, HYDIN, MAL, MGARP, MT-CO1, MT-ND4, NCSTN, NDUFS3, NDUFS4, NME5, PAFAH1B1, RAN, SH3GL1, SPEF2, SPINK5, TCTN1, TUBA1A, TUBB2A, UGP2, UQCRQ, VIM, VTN, WDR37, YWHAE, YWHAH, YWHAQ	Central nervous system development	
LIN (RM) vs (Pe + BS + BP)	p = 0.05	DCTN5, FKBP4, DYNLL2, STIP1, DYNLL1, PTGES3, HSP90AA1, TUBB4B, TUBA1A, HSPA8, TUBA4A, TUBA3E, CAPZA3, CAPZB, DNAJA2, DNAJA4, TUBB2A, ACTR1A		HSP90 chaperone cycle for steroid hormone receptors SHR in the presence of ligand
VSL (RM) vs (BS + Pe + BP)	p = 0.005	COX7C, COX7B, MT-CO1, YWHAH, YWHAG, MT-CO2, COX6A1, TXN, COX5A, YWHAB, NDUFA4, YWHAE, COX6C, PRDX1, YWHAQ, YWHAZ, GPI, COX4I1, COX7A2L		TP53 regulates metabolic
		PDK3, SLC25A11, SLC25A13, DLAT, HK3, PFKP, G6PC3, AKR1B1, SLC2A3, PDHB, PDHA2, GPI, DYRK2, ALDOC, MDH2, GAPDH, ENO1, PGK2, TPI1, PGAM1, MPI, GSK3A, HEXB, MDH1, GOT1, HEXA, SLC25A12, PGD, USP7, GSTO2, SORD, OTOGL, PGP	Monosaccharide metabolic process	
VCL (RM) vs (Pe)	p = 0.05	MSLN, AHSG, FN1, FGG, CKAP4, FGA, C3, SERPINC1, APOA1, PLG, F2, TF, ALB, HSP90B1, P4HB		Regulation of insulin like growth factor IGF transport and uptake by insulin like growth factor binding proteins IGFBPS
STR (RM) vs (BS)	p = 0.05	DYNLL1, TUBA3E, FKBP4, TUBB2A, DCTN5, DYNLL2, DNAJA4, DNAJA2, STIP1, PTGES3, HSPA8, ACTR1A, CAPZB, TUBB4B, HSP90AA1, CAPZA3, TUBA4A, TUBA1A		HSP90 chaperone cycle for steroid hormone receptors SHR in the presence of ligand
Vo (Pe) vs (RM)	p = 0.05	DYNLL1, TUBA3E, FKBP4, TUBB2A, DCTN5, DYNLL2, DNAJA4, DNAJA2, STIP1, PTGES3, HSPA8, ACTR1A, CAPZB, TUBB4B, HSP90AA1, CAPZA3, TUBA4A, TUBA1A		HSP90 chaperone cycle for steroid hormone receptors SHR in the presence of ligand
		DYNLL1, RAB1B, TUBA3E, TUBB2A, ANK3, DCTN5, DYNLL2, ACTR1A, CAPZB, TUBB4B, ARF1, ARF4, SAR1B, CAPZA3, TUBA4A, TUBA1A		ER to GOLGI anterograde transport
		GOLGA4, DYNLL1, RAB1B, TUBA3E, RAB6A, TUBB2A, DCTN5, CENPE, DYNLL2, ACTR1A, KIF2B, CAPZB, TUBB4B, KIF9, ARF1, ARF4, CAPZA3, TUBA4A, PAFAH1B1, TUBA1A, VAMP3		Intra GOLGI AND retrograde GOLGI to ER traffic
		DYNLL1, RAB1B, TUBA3E, TUBB2A, ANK3, DCTN5, DYNLL2, ACTR1A, CAPZB, TUBB4B, ARF1, ARF4, CAPZA3, TUBA4A, TUBA1A		COPI mediated anterograde transport

The protein datasets of breeds with score 2 (highest performances) and score −2 (lowest performances) were compared by GSEA in terms of molecular pathways (reactome) and biological processes (GOBP). In each comparison over-represented biological processes (GOBP) and pathways (REACTOME) are reported along with the p-value applied in each enrichment map. Number of semen samples analyzed: BP, N = 4; BS, N = 4; MB, N = 4; Pe, N = 4; RM, N = 4.

#### Enrichment map analysis revealed significantly enriched molecular processes (p < 0.05), including.

HPS90 chaperone cycle for steroid hormone receptors (SHR), TP53 transcriptional regulation, insulin-like growth factor transport and uptake (IGFBPS) and Golgi and endoplasmic reticulum (ER) trafficking, as well as retrograde and COPI anterograde transport.

**Fig 3:**
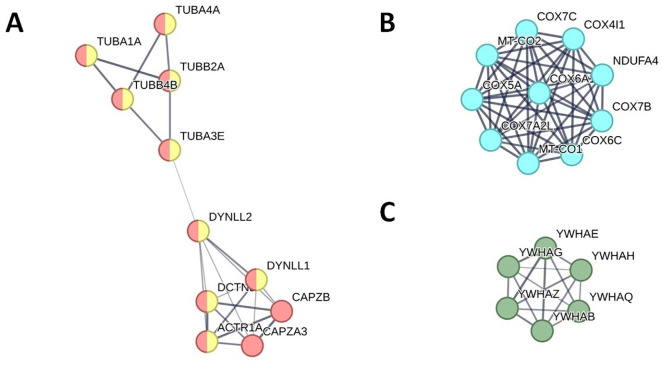
String analysis. Network analysis by String of the proteins involved in A) the HSP90 chaperone cycle for the steroid hormone receptors (SHR) pathway. Cytoskeletal and microtubule-associated proteins are indicated in red and yellow, respectively; B) and C) the TP53 transcriptional regulation pathway. Proteins involved in complex IV of cellular respiration are indicated in cyan, while those involved in the regulation of intracellular calcium levels are shown in green. Line thickness indicates the strength of data support. The minimum required interaction score is medium (0.4), hiding disconnected nodes.

The Hsp90 chaperone cycle for SHR was enriched in breeds exhibiting superior WOB, PMS, SMI, LIN, STR and semen volume, while it was decreased in those with better ALH. Related cytoskeletal proteins were identified (Table 5). STRING network analysis reveals several proteins involved in this pathway (ACTR1A, CAPZA3, CAPZB, DCTN5, DYNLL, DYNLL2, TUBA1A, TUBA3E, TUBA4A, TUBB2A and TUBB4B), most of which are cytoskeletal components that contribute to the microtubule structure (Tables 4 and 5, Fig 3A).

Proteins involved in Golgi and ER trafficking, including both retrograde and COPI anterograde transport, were also enriched in breeds with better SMI and higher ejaculate volume, as shown in Tables 4 and 5. Additional microtubule-associated proteins (CENPE, KIF2B, KIF9, PAFAH1B1) were also identified within these pathways (Tables 5).

Proteins related to the TP53 transcriptional regulation pathway were found to be increased in breeds with higher VSL.

The bioinformatic analysis also highlights an enrichment in proteins involved in Complex IV of cellular respiration, namely COX4I1, COX5A, COX6A1, COX6C, COX7A2L, COX7B, COX7C, MT-CO1, MT-CO2 and NDUFA4, (Tables 4 and 5, Fig 3B). Proteomic analysis reveals that some of the proteins identified in this pathway (YWHAB, YWHAE, YWHAG, YWHAH, YWHAQ and YWHAZ) are involved in the follicular development during reproductive phases transition [19,52,53] and play a crucial role in regulating intracellular calcium levels (Tables 4 and 5, Fig 3C).

Lastely, proteins associated with insulin-like growth factors (IGFs) transport and uptake by their respective binding proteins (IGFBPs) were upregulated in breeds exhibiting better VAP and VCL parameters.

To further support the shotgun proteomic findings and reinforce the biological relevance of selected proteins associated with semen quality traits, a targeted proteomic analysis using Parallel Reaction Monitoring (PRM) was carried out on few proteins selected based on their differential abundance or involvement in key biological pathways identified through enrichment analysis (Table 5), namely: dynactin subunit 5 (DCTN5), NFS attachment protein alpha (NAPA), sulfurtransferase (MPST), and glycerol-3-phosphate phosphatase (PGP).

NFS attachment protein alpha (NAPA) emerges as a key mediator of vesicle trafficking and membrane remodeling processes. Its functional relevance is particularly evident during spermiogenesis, where its contributes to acrosome biogenesis and plasma membrane reorganization. NAPA is also associated with pathways involved in head and central nervous system development, suggesting broader roles in cellular differentiation and structural organization. Targeted analysis confirmed its identification in MB breed (S7 Table, S1B Fig) consistent with the breed’s distinct kinetic profile. These findings support the hypothesis that efficient intracellular transport mechanisms may contribuite to the superior sperm motility obsterved in MB males [41].

Sulfurtransferase (MPST) is also listed among the proteins involved in central nervous system development. MPST is involved in redox homeostasis and the production of hydrogen sulfide, both of which are critical for cellular stress responses and mitochondrial function. While traditionally associated with neural development, emerging evidence supports a functional role for MPST in sperm, particularly in the regulation of mitochondrial dynamics and oxidative balance, factors known to influence sperm quality and motility [50,51]. The targeted proteomic analysis confirmed its exclusive presence in the BP breed (S8 Table, S1C,D Figs), a breed characterized by lower overall motility. This finding suggests a potential compensatory mechanism aimed at maintaining sperm structural integrity rather than enhancing motility in this genetic background [51].

Dynactin subunit 5 (DCTN5) was selected among the proteins involved in the HPS90 chaperone cycle for steroid hormone receptors SHR pathway according to the GSEA analysis reported in Table 5. DCTN5 is a component of the dynactin complex and plays a critical role in microtubule-based transport and cytoskeletal organization, processes essential for proper sperm flagellum function and plays a potential role in hormone-mediated regulation of reproductive functions [35,36]. The targeted analysis confirm that the protein is uniquely identified in the RM breed (S11 Table, S1A Fig) which is characterized for its superior sperm motility, particularly in PMS, VSL, LIN, and STR strengthening the DCTN5 involvement in motility efficiency.

Glycerol-3-phosphate phosphatase (PGP) is a component of the monosaccharide metabolic pathway reported in Table 5 highlighting the importance of carbohydrate metabolism in sperm energetics, particularly in the regulation of glucose- and glycerol-derived pathways critical for ATP production during motility. In keeping with the untargetd results (S9 Table), the targeted proteomic analysis detected the protein only in the BS breeds (S1E Fig), which exhibit intermediate motility traits in line with the importance of energy utilization pathways for the sperm motility efficiency.

Overall, the targeted analysis reinforce the untargeted results and the bioinformatic findings which suggest that these proteins act as biological effectors within key signaling and metabolic pathways linked to sperm quality variability in avian species and are, therefore, potentially important in biomarker discovery, fertility screening, and optimization of cryopreservation strategies for poultry genetic resource conservation.

## Discussion

Conservation of animal genetic resources, particularly poultry, is essential to preserve biodiversity and ensure sustainable livestock production under changing environmental conditions. In this study, we characterized the sperm proteome of five Italian chicken breeds and investigated the relationship between proteomic variability and semen quality. Our findings provide new insights into breed-specific protein expression and its association with critical parameters such as sperm motility kinematics, and membrane integrity, demonstrating that semen quality is strongly influenced by breed.

Semen quality differed significantly among breeds, with no direct association between quantitative and qualitative sperm production. While TMS was consistent across all breeds, suggesting uniform sperm maturation within the vas deferens, other sperm characteristics varied significantly. Pe roosters produced the highest semen volume and concentration but their sperm exhibited reduced motility efficiency, as reflected by the lowest VCL, VSL, and VAP values. These results suggest that increased sperm production does not necessarily translate into improved sperm function. Conversely, RM and MB roosters showed the opposite trend, producing lower semen volume and concentration but superior motility profiles. RM males showed the highest VSL, VAP, LIN, STR, and WOB values, indicative of progressive and linear sperm movement, whereas MB roosters showed a high SMI and favorable kinematic traits, supporting the functional quality of their ejaculates. BP and BS showed intermediate profiles, highlighting breed-specific strategies and a potential trade-off between ejaculate volume and sperm motion efficiency, which should be considered in conservation and breeding programs.

Proteomic profiling further supported these functional observations, revealing a high number of identified proteins per breed (ranging from 848 in RM to 989 in BS) and clear clustering by breed, as evidenced by PCA. In RM, the high motility performance was associated with proteins involved in energy metabolism and flagellar function. PKLR, a key enzyme in glycolysis, plays a crucial role in energy production required for flagellar beating [35], while the proteins TMEM107 and DCTN5 contribute to glucose metabolism, sperm flagellum morphology, and spermatid growth [36–38]. Together, these proteins enhance energy availability and structural support, aligning with RM’s superior motility traits. Similarly, MB showed strong kinematic performance, likely linked to proteins such as RPS8, previously identified as a marker of male fertility [39], and PPP3CA, whose inhibition prevents fertility in mice [40]. These proteins, together with others involved in carbohydrate metabolism and mitochondrial transport, suggest that MB relies on efficient metabolic pathways to fuel sperm motility and support fertility. In contrast, Pe showed lower sperm motility but higher SMI. Its proteomic profile indicates a prioritization of membrane integrity over motility, with proteins such as CRAT and PRKX, playing roles in chromatin quality, lipid metabolism and cell signaling [42,43]. We can hypothesize that this strategy may improve membrane stability and sperm viability despite reduced kinematic performance. BS breed shows a balanced profile with good motility and membrane integrity. UCHL3, a protein associated with sperm count, motility, and fertilization efficiency in humans [44], was detected in this breed and likely supports its reproductive performance. On the other hand, BP was characterized by proteins such as PSAP, TFAM, and UBE2V1. PSAP is linked to fertility through glycosphingolipid catabolism [45]. TFAM regulates spermatogenesis and mitochondrial function. [47–49] and UBE2V1 is essential for sperm maturation, with knockout studies showing infertility in mice lacking this [46]. Their combined presence suggests that BP maintains sperm motility and membrane integrity through coordinated metabolic and structural processes.

All these findings highlight the importance of breed-specific proteins in determining semen quality, a concept further supported by studies in other species and strengthened by pathway enrichment analysis. The Hsp90 chaperone cycle for steroid hormone receptors (SHR) was enriched in breeds with superior WOB, PMS, SMI, LIN, STR and seminal volume consistent with its role in male reproductive development and fertility [67,84,85]. Network analysis revealed that several proteins in this pathway (ACTR1A, CAPZA3, CAPZB, DCTN5, DYNLL, DYNLL2, TUBA1A, TUBA3E, TUBA4A, TUBB2A and TUBB4B) are cytoskeletal components essential for microtubule organization, linking cytoskeletal integrity to fertility [55,69,52,70,86]. Several of these proteins are also involved in Golgi and ER trafficking-related pathways, processes required for spermatogenesis and acrosome formation [87–90]. Additional microtubule-associated proteins, such as CENPE, KIF2B, KIF9 and PAFAH1B1 further highlight the importance of intracellular trafficking for sperm function [71–73,91]. Other enriched pathways included TP53 transcriptional regulation, which was associated with improved VSL. TP53 is crucial for maintaining genomic integrity in germ cells for regulating early embryonic development, and polymorphisms in this gene have been significantly associated with male infertility [92,93]. Pathways related to Complex IV of cellular respiration (COX4I1, COX5A, COX6A1, COX6C, COX7A2L, COX7B, COX7C, MT-CO1, MT-CO2 and NDUFA4) were also prominent, consistent with the well-established role of mitochondrial activity in supporting sperm motility [74,94]. Additionally, proteomic analysis reveals that some of the proteins identified in this pathway (YWHAB, YWHAE, YWHAG, YWHAH, YWHAQ and YWHAZ) are involved in the follicular development during reproductive phases transition [20,75,76] and play a crucial role in regulating intracellular calcium levels, which is a key factor in reproductive processes [95]. Finally, the transport and uptake of insulin-like growth factors (IGFs) by their respective binding proteins (IGFBPs) were increased in breeds exhibiting better VAP and VCL. Altered IGF signaling has been linked to sperm motility in humans and animals [77,78], suggesting a conserved role across species. In vitro, IGF-I supplementation has been shown to improve motility in sperm cattle, stallion, and buffalo [78], reinforcing its relevance for poultry reproduction.

Overall, these findings demonstrate that breed-specific proteomic signatures underlie the observed variability in semen quality traits. The identified proteins and pathways act as biological effectors linking molecular mechanisms to phenotypic performance and may serve as candidate markers for evaluating male reproductive potential. Such insights are essential for the efficient conservation and sustainable management of poultry genetic resources, providing a molecular basis to guide strategies that preserve both biodiversity and reproductive performance.

## Conclusions

This investigation represents the first proteomic comparison of semen in five local Italian chicken breeds at risk of extinction. The results highlight significant inter-breed variability in semen quality and sperm proteome composition, underscoring the value of a comparative approach to understand the molecular basis of breed-specific sperm function. Proteomic analysis revealed that each breed exhibits a unique set of sperm proteins associated with specific semen quality parameters. Notably, differences were identified in molecular pathways related to energy metabolism, membrane integrity, and sperm motility suggesting breed-specific regulation of the molecular mechanisms underpinning semen traits. These findings emphasize the importance of studying local breeds as models for exploring biological diversity and reproductive specialization in poultry, also contributing to a deeper understanding of functional variation among breeds and supporting the development of informed strategies for the conservation and sustainable use of avian genetic resources.

## Supporting information

S1 Table Proteins identified in sperm cells of Mericanel della Brianza (MB) chicken breed, by a shotgun label-free proteomic approach. Protein IDs and the corresponding gene name are indicated for each identified protein (N = 4).
(XLSX)

S2 Table Proteins identified in sperm cells of Bionda Piemontese (BP) chicken breed, by a shotgun label-free proteomic approach. Protein IDs and the corresponding gene name are indicated for each identified protein (N = 4).
(XLSX)

S3 Table Proteins identified in sperm cells of Bianca di Saluzzo (BS) chicken breed, by a shotgun label-free proteomic approach. Protein IDs and the corresponding gene name are indicated for each identified protein (N = 4).
(XLSX)

S4 Table Proteins identified in sperm cells of Pepoi (Pe) chicken breed, by a shotgun label-free proteomic approach. Protein IDs and the corresponding gene name are indicated for each identified protein (N = 4).
(XLSX)

S5 Table Proteins identified in sperm cells of Robusta Maculata (RM) chicken breed, by a shotgun label-free proteomic approach. Protein IDs and the corresponding gene name are indicated for each identified protein (N = 4).
(XLSX)

S6 Table Reproducibility of the protein extraction method. Left block: Number of identified proteins for each biological replicate belonging to a group (Bionda Piemontese (BP), Bianca di Saluzzo (BS), Mericanel della Brianza (MB), Pepoi (Pe) and Robusta Maculata (RM); Right block: Pearson correlation coefficient of protein intensities in each biological replicate is indicated. Number of semen samples analyzed: BP, N = 4; BS, N = 4; MB, N = 4; Pe, N = 4; RM, N = 4.
(XLSX)

S7 Table Proteins exclusively detected in sperm cells of Mericanel della Brianza (MB) chicken breed, by a shotgun label-free proteomic approach. Comparison Mericanel della Brianza (MB) *vs* Bionda Piemontese (BP) *vs* Bianca di Saluzzo (BS) *vs* Pepoi (Pe) *vs* Robusta Maculata (RM). Protein IDs and the corresponding gene name are indicated for each identified protein (N = 4).
(XLSX)

S8 Table Proteins exclusively detected in sperm cells of Bionda Piemontese (BP) chicken breed, by a shotgun label-free proteomic approach. Comparison Mericanel della Brianza (MB) *vs* Bionda Piemontese (BP) *vs* Bianca di Saluzzo (BS) *vs* Pepoi (Pe) *vs* Robusta Maculata (RM). Protein IDs and the corresponding gene name are indicated for each identified protein (N = 4).
(XLSX)

S9 Table Proteins exclusively detected in sperm cells of Bianca di Saluzzo (BS) chicken breed, by a shotgun label-free proteomic approach. Comparison Mericanel della Brianza (MB) *vs* Bionda Piemontese (BP) *vs* Bianca di Saluzzo (BS) *vs* Pepoi (Pe) *vs* Robusta Maculata (RM). Protein IDs and the corresponding gene name are indicated for each identified protein (N = 4).
(XLSX)

S10 Table Proteins exclusively detected in sperm cells of Pepoi (Pe) chicken breed, by a shotgun label-free proteomic approach. Comparison Mericanel della Brianza (MB) *vs* Bionda Piemontese (BP) *vs* Bianca di Saluzzo (BS) *vs* Pepoi (Pe) *vs* Robusta Maculata (RM). Protein IDs and the corresponding gene name are indicated for each identified protein (N = 4).
(XLSX)

S11 Table Proteins exclusively detected in sperm cells of Robusta Maculata (RM) chicken breed, by a shotgun label-free proteomic approach. Comparison Mericanel della Brianza (MB) *vs* Bionda Piemontese (BP) *vs* Bianca di Saluzzo (BS) *vs* Pepoi (Pe) *vs* Robusta Maculata (RM). Protein IDs and the corresponding gene name are indicated for each identified protein (N = 4).
(XLSX)

S1 Fig Parallel reaction monitoring (PRM) assay for detection of dynactin subunit 5 (DCTN5), NFS attachment protein alpha (NAPA), sulfurtransferase (MPST), and glycerol-3-phosphate phosphatase (PGP) in RM, MB, BP and BS chicken breed, respectively. Extracted-ion chromatogram (XIC) of the transitions observed for the peptide: A) 1-MELSEMLYNK-10 from dynactin subunit 5 (DCTN5), as measured by PRM in Robusta Maculata (RM); B) 272- LDQWLTTMLLR-282 from NFS attachment protein alpha (NAPA), as measured by PRM in Mericanel della Brianza (MB); C-D) 53-HIPGAVFFDIDQCSDR-68 and 165-TYEDILDNLDSHR-177 from sulfurtransferase (MPST), as measured by PRM in Bionda Piemontese (BP); E) 38- GEAALSGAPAALGR-51, as measured by PRM in Bianca di Saluzzo (BS).
(TIF)
