# Supplementary figures and images for: Relation between proteome characterization and semen quality in Italian chicken breeds
Source: PLoS One. 2025 Oct 8;20(10):e0333802. doi: 10.1371/journal.pone.0333802 (PMC12507248; doi:10.1371/journal.pone.0333802)

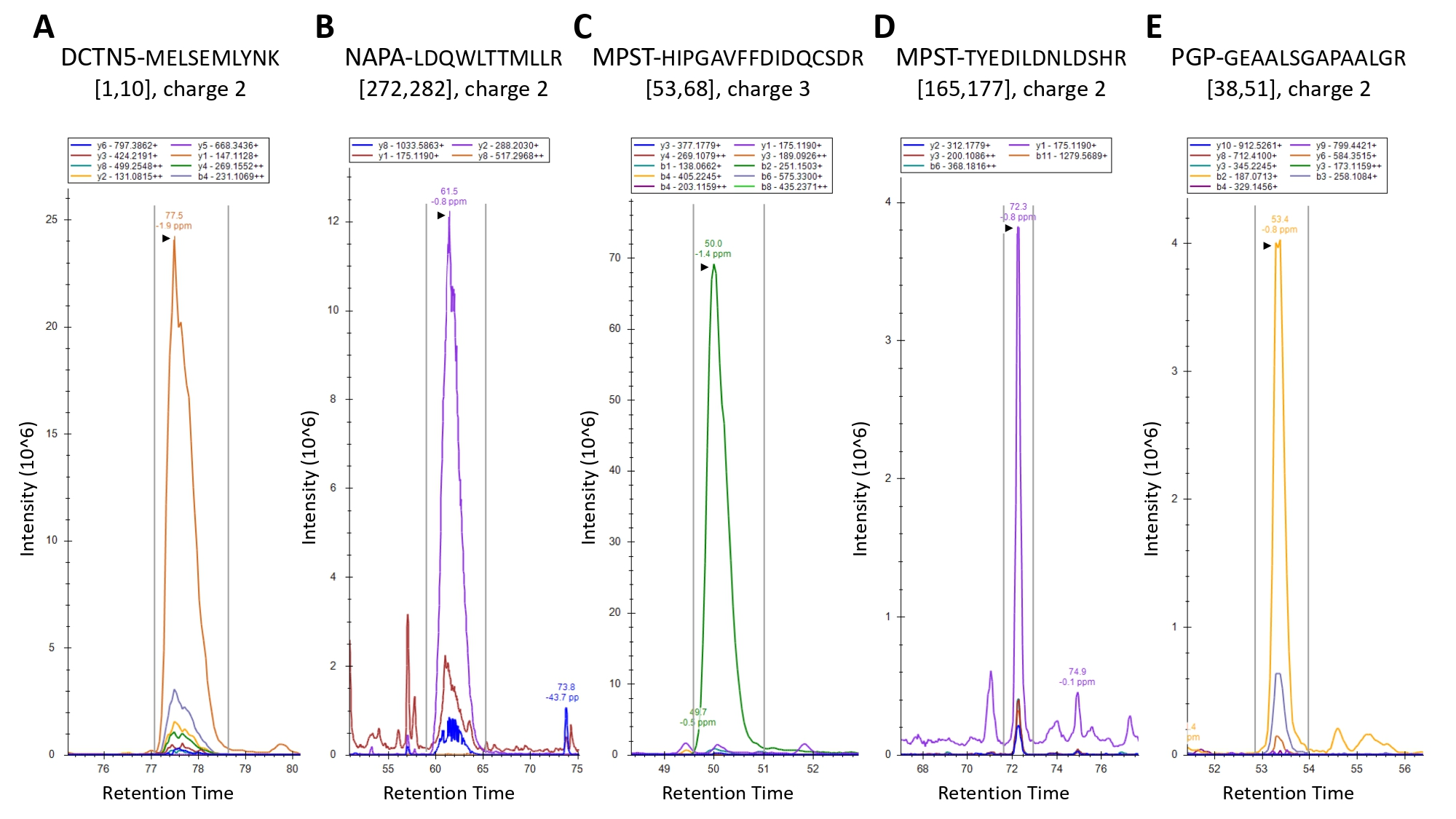

Supplement: S1 Fig — Extracted-ion chromatogram (XIC) of the transitions observed for the peptide: A) 1-MELSEMLYNK-10 from dynactin subunit 5 (DCTN5), as measured by PRM in Robusta Maculata (RM); B) 272- LDQWLTTMLLR-282 from NFS attachment protein alpha (NAPA), as measured by PRM in Mericanel della Brianza (MB); C-D) 53-HIPGAVFFDIDQCSDR-68 and 165-TYEDILDNLDSHR-177 from sulfurtransferase (MPST), as measured by PRM in Bionda Piemontese (BP); E) 38- GEAALSGAPAALGR-51, as measured by PRM in Bianca di Saluzzo (BS). (TIF) [file pone.0333802.s012.tif]
